# Supplementary figures and images for: Comparison between the Amount of Environmental Change and the Amount of Transcriptome Change
Source: PLoS One. 2015 Dec 14;10(12):e0144822. doi: 10.1371/journal.pone.0144822 (PMC4678807; doi:10.1371/journal.pone.0144822)

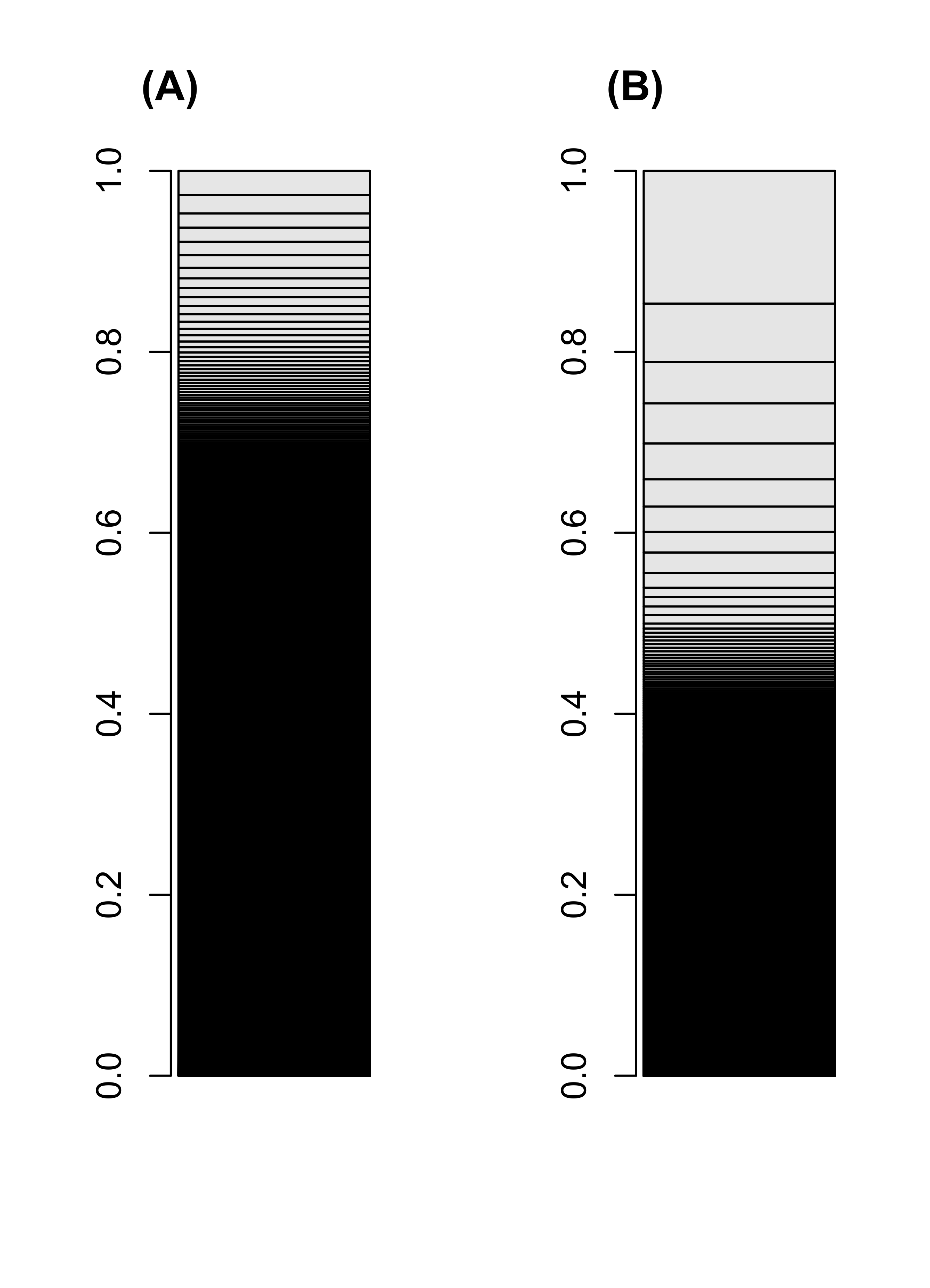

Supplement: S1 Fig — Although more than 14,000 genes are included in these bar charts, most are invisible and are included in the black regions. Transcriptomes of fat-body cells cultured for 10 hours in MGM-450 insect medium supplemented with (A) 0.25 mM and (B) 2.5 mM cis-permethrin, after cultivation for 80 hours in cis-permethrin–non-supplemented MGM-450 insect medium. (TIF) [file pone.0144822.s001.tif]

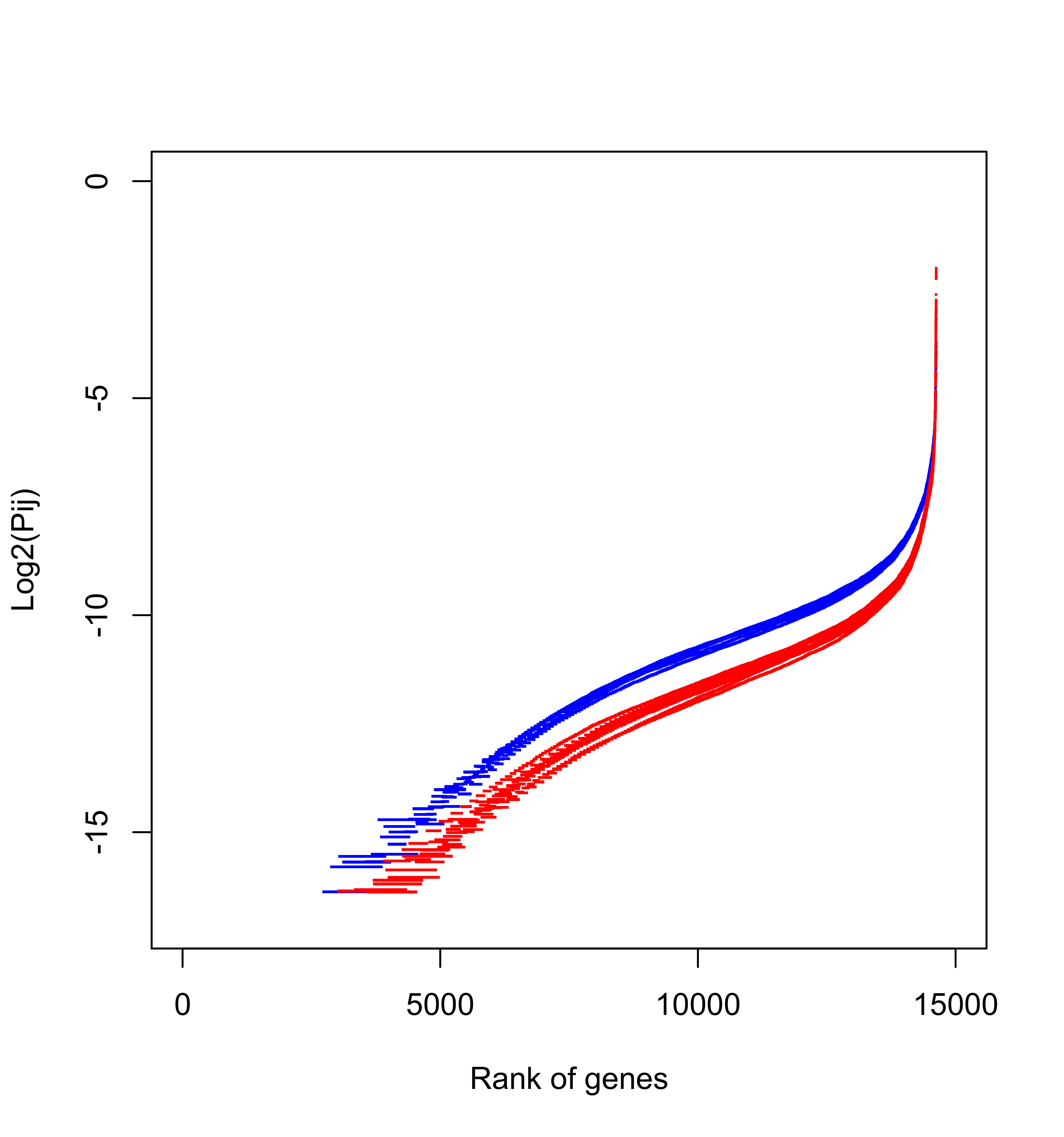

Supplement: S2 Fig — Genes were sorted in order of log2(P ij). Transcriptomes with high diversity (0 and 0.25 mM phenobarbital experimental section) are plotted as blue dots. Those with low diversity (1.0, 2.5, and 12.5 mM phenobarbital experimental section) are plotted as red dots. (TIF) [file pone.0144822.s002.tif]

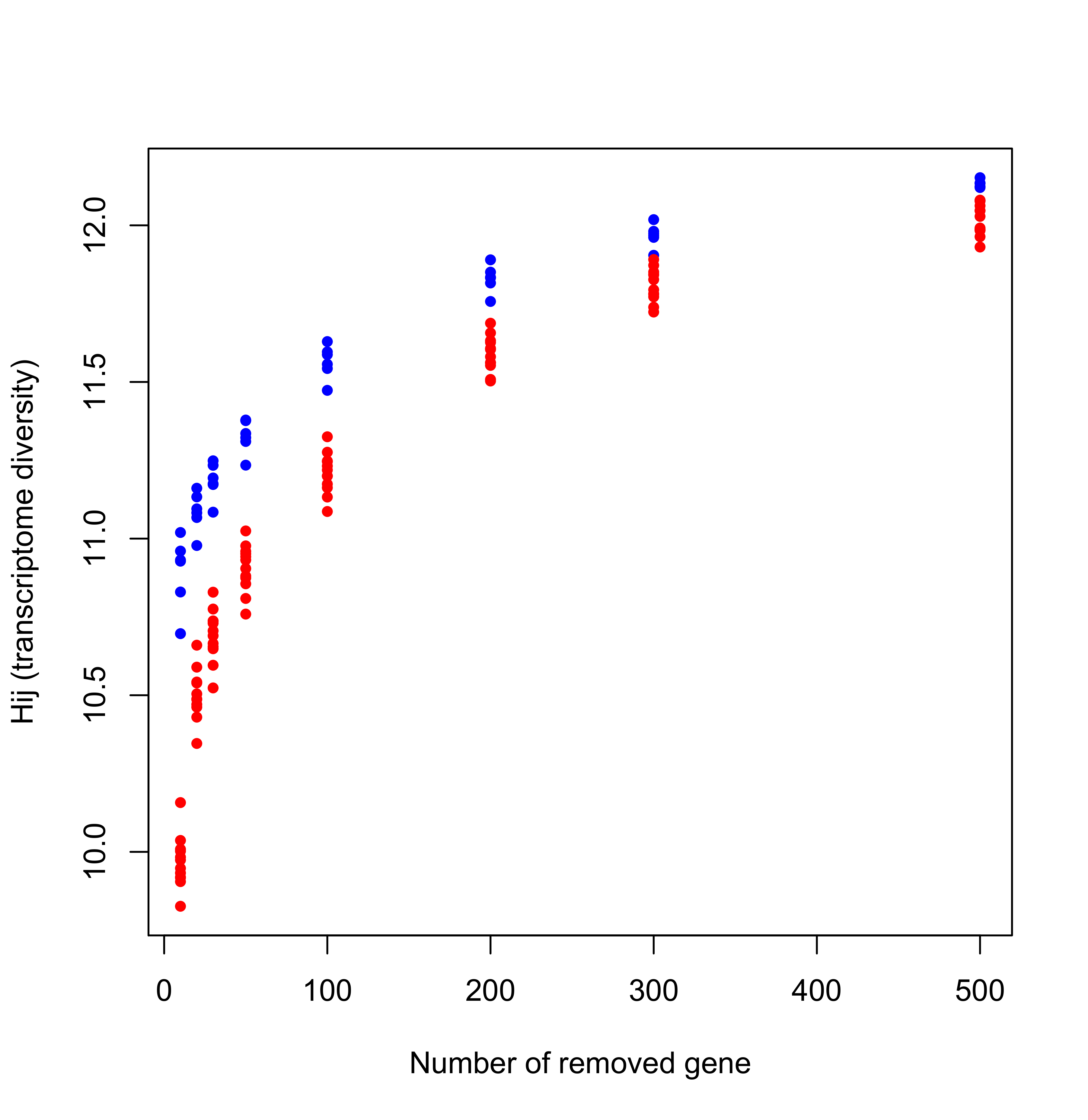

Supplement: S3 Fig — The diversity of transcriptomes lacking the top 10, 20, 30, 50, 100, 200, 300, and 500 most-expressed genes was estimated. Transcriptomes with high diversity (0 and 0.25 mM phenobarbital experimental section) are plotted as blue dots. Those with low diversity (1.0, 2.5, and 12.5 mM phenobarbital experimental section) are plotted as red dots. (TIF) [file pone.0144822.s003.tif]
